# Fluoride releasing in polymer blends of poly(ethylene oxide) and poly(methyl methacrylate)

**DOI:** 10.3389/fchem.2024.1356029

**Published:** 2024-02-09

**Authors:** Tianxiao Wang, Menghong Li, Ziyan Gu, Chengjuan Qu, Jonas Segervald, Roushdey Salh, Thomas Wågberg, Jia Wang, Wen Kou

**Affiliations:** ^1^ Department of Odontology, Umeå University, Umeå, Sweden; ^2^ Department of Physics, Umeå University, Umeå, Sweden

**Keywords:** dental materials, polymethyl methacrylate, polyethylene oxide, fluoride ion release, polymer blend

## Abstract

**Introduction:** Polymethyl methacrylate is a polymer commonly used in clinical dentistry, including denture bases, occlusal splints and orthodontic retainers.

**Methods:** To augment the polymethyl methacrylate-based dental appliances in counteracting dental caries, we designed a polymer blend film composed of polymethyl methacrylate and polyethylene oxide by solution casting and added sodium fluoride.

**Results:** Polyethylene oxide facilitated the dispersion of sodium fluoride, decreased the surface average roughness, and positively influenced the hydrophilicity of the films. The blend film made of polymethyl methacrylate, polyethylene oxide and NaF with a mass ratio of 10: 1: 0.3 showed sustained release of fluoride ions and acceptable cytotoxicity. Antibacterial activity of all the films to Streptococcus mutans was negligible.

**Discussion:** This study demonstrated that the polymer blends of polyethylene oxide and polymethyl methacrylate could realize the relatively steady release of fluoride ions with high biocompatibility. This strategy has promising potential to endow dental appliances with anti-cariogenicity.

## 1 Introduction

Dental caries in permanent teeth affect 2.3 billion people, with a global prevalence of 35% for all ages (James et al., 2018; Peres et al., 2019; Shi et al., 2022). For the prevention of dental caries, fluoride delivery, for instance, sodium fluoride (NaF), has become an active and popular method for the prevention of dental caries (Amina et al., 2019; Nagireddy et al., 2019). The anticariogenic mechanism of fluoride can be simply summarized as follows: 1) it inhibits tooth demineralization, 2) it promotes enamel remineralization, and 3) it interferes with bacterial metabolism and plaque formation (Featherstone, 1999; Wiegand et al., 2007; Nassar and Brizuela, 2023).

Polymethyl methacrylate (PMMA) is a polymer that is widely used in everyday applications. These appliances, for instance, occlusal splints or orthodontic retainers, are usually recommended to be worn in the mouth for a minimum of 8 h per day (Castle et al., 2019; Zhang L. et al., 2021; Dogramaci and Littlewood, 2021; Ferrillo et al., 2022; Nazarov Olimjon et al., 2022) and have direct contact with teeth surfaces and saliva (Thaweboon and Thaweboon, 2019; Raszewski et al., 2021; Potewiratnanond et al., 2023). This makes PMMA-based dental appliances the desirable fluoride-ion-releasing vehicles. Since administration of high doses of fluoride may increase the risk of dental and skeleton fluorosis (Ayoob and Gupta, 2006; Ding et al., 2020; Dong et al., 2021), developing a model that can realize a sustained release of fluoride at a low concentration level is particularly appealing for practical applications.

To realize a controllable fluoride ion release with PMMA-based dental materials, the main challenge is that inorganic salt NaF is hydrophilic (Yao et al., 2021) and only dissolves in water and slightly dissolves in a few polar aprotic solvents; whereas PMMA is rich in hydrophobic functional groups and its insulator property does not easily allow the movement of ions within its bulk (Liu et al., 2020; Sui et al., 2021). Consequently, a significant phase-separation will form in the mixture of PMMA and NaF. It can also result in, for example, fluoride ions barely distributed in PMMA bulk, instead agglomerated as large crystalline chunks within certain domains, on the surface of or inside of PMMA bulk. Another consequence is that these fluoride ions blended in PMMA bulk cannot efficiently release due to the lack of a releasing pathway.

In this context, introducing another polymer, which can be homogeneously mixed with both PMMA and NaF might tackle this problem. Polyethylene oxide (PEO) is a well-known water-soluble polymer, composed of unit ethylene oxide. Due to its high biocompatibility, low toxicity, easy processability, and mild amphiphilicity, PEO is widely used in skin care products, toothpaste, food, drinks, and drug release systems (Emerine and Chou, 2022). But the most important property is that PEO can form complexes with cations and its polymer chain possesses high flexibility, which can promote ion transport. Therefore, PEO has been intensively studied as the polymer host material in solid polymer electrolytes (Knop et al., 2010; Devaux et al., 2012; Xue et al., 2015). Taking these advantages into account, we anticipate that introducing PEO into PMMA bulk can improve the NaF dispersion in polymer bulk. It can also enhance the fluoride release by providing a fluoride-ion-motion pathway along its molecular chains.

In this study, we prepared PMMA/PEO/NaF blend films by solution casting to investigate the effect of PEO on fluoride ion release behavior. The blend films were prepared via the identical protocol but composed of PMMA: PEO and NaF with different mixing mass ratios, ranging from 10: 0: 0.3 to 10: 3: 0.3. The surface and cross-sectional morphologies of these polymers blend films were investigated by means of electronic microscopy. The element composition and bonding information of these blend films were analyzed. The surface roughness and contact angle were also measured and compared. Fluoride ion releasing behaviors of all polymer films were monitored by using a pH/ion selective electrode (ISE) meter to detect the fluoride ion content released by the films in water over 14 days, and the cumulative release concentration and proportion were calculated accordingly. Furthermore, the cytotoxicity of the blend films was tested to evaluate the *in vitro* biocompatibility, and the antibacterial property was tested with *Streptococcus mutans* (*S. mutans*).

## 2 Materials and methods

### 2.1 Materials

Meliodent^®^ Heat Cure clear (Kulzer GrmbH, Hanau, Germany), a commercially available PMMA based material for manufacturing dental occlusal splints, was used to manufacture polymer blend films, which concludes powder and liquid. The powder is methyl methacrylate copolymer, and the liquid consists of methyl methacrylate (above 90%), 1,4-butandiodimethacrylate (1%–5%) and p-Mentha-1,4-diene (less than 0.25%). PEO with a viscosity-average molecular weight of 6.0 × 10^5^ g/mol (Sigma-Aldrich Chemie GmbH, Schnelldorf, Germany), sodium fluoride (NaF, ≥99%, Sigma-Aldrich Chemie GmbH, Schnelldorf, Germany) and acetone (GPR Rectapur, VWR International, France) were used to prepare the samples. All reagents were used as received without further purification.

The water used for sample preparation and testing was gained from Milli-Q^®^ EQ 7000 Ultrapure Water Purification System (Merck KGaA, Darmstadt, Germany).

### 2.2 Preparation of samples

The films were prepared by solution casting. 1, 2 and 3 g of PEO were mixed with 0.3 g of NaF, respectively, and the mixtures were stirred in 40 mL of acetone at 50°C for 2 h with a speed of 300 rpm (DLABTM MS-H280-Pro Hot Stirrer, Beijing, China) to form a homogenous mixture.

7.5 g of powder from the Heat Cure clear kit was blended with 2.5 g of liquid from the same kit, placed in a ventilation closet for 10 min, and the mixture was manually separated into millimeter-sized flakes. PMMA flakes were then mixed with the NaF/PEO mixtures. 120 mL acetone was then added into the resultant mixtures to reduce the viscosity. The mixture was stirred at 50°C with a speed of 300 rpm until an even and homogeneous slurry was received.

The slurry was casted in a Petri dish and placed in an oven and dried at 50°C for 1 h. The obtained films were named according to the mass ratio of PEO containing in them. Specifically, the films containing 1, 2, and 3 g of PEO were named the PEO-10, the PEO-20 and the PEO-30 group, respectively. The films without PEO were named the PEO-0 group. The composition of each group was listed in Supplementary Table S1.

### 2.3 Scanning electron microscope (SEM) observation

After completely drying out, the as-prepared films were peeled off from the Petri dish and cut into small samples (10 mm × 10 mm). The surface morphologies of the samples were observed by SEM (Carl ZeissTM Evo LS 15, Oberkochen, Germany) at 50 Pa using secondary electron (VPSEE G4) and probe current of 500 pA. Elemental composition analysis was performed using energy dispersive spectrometer (EDS, Oxford InstrumentsTM X-Max 80 mm^2^, High Wycombe, United Kingdom) at an accelerating voltage of 20 kV and a probe current of 700 pA.

### 2.4 Phase and composition analysis

Topography and phase measurements were performed on a Veeko multimode atomic force microscope (AFM), and the images were taken in tapping mode on an area of 3 × 3 μm in ambient air condition.

A Panalytical X’pert X-ray diffractometer in reflection mode with CuKα radiation was used to record the X-Ray diffraction (XRD, PANalytical B.V., Almelo, the Netherlands) of the samples. The XRD patterns were recorded in an angle range from 5° to 72° with a step size of 0.007°, a time per step of 100 s, and a total of 90 min per scan.

The Fourier-transform infrared (FTIR) spectra of the samples were collected with FTIR spectrometer (Bruker Vertex 80 vacuum, Bruker Optik GmbH, Ettlingen, Germany). The samples were fixed and detected by a diamond probe, in transmission mode.

### 2.5 Surface roughness measurement

The average surface roughness was measured with a surface profilometer (50 mm Intra 2, Taylor Hobson Ltd., Leicester, England). The probe moved at a speed of 0.5 mm/s, and the tested length was 5 mm. The scanning area was 25 mm^2^. The data were processed with a Gaussian filter and the surface average roughness value was obtained in the form of Ra. Five samples were measured for each group.

### 2.6 Static contact angle measurement

The static contact angle was measured with a theta lite optical tensiometer (Biolin Scientific, Västra Frölunda, Sweden). A droplet of 4 μL water was dropped on each group, and the images were taken with the equipped camera. The value of the contact angle was fitted with software, and three samples were measured for each group.

### 2.7 Fluoride ion release tests

Films were cut into rectangular samples measuring 30 mm × 15 mm and then the samples were immersed separately in 5 mL of water in 15 mL centrifuge conical tubes with caps (Sarstedt Inc., United States) for 2 h before the tests to eliminate the effect of rapid release of NaF clusters on the surface. During the tests, the samples were still immersed in 5 mL of water. The immersion water was taken out and placed in clean centrifuge conical tubes at scheduled time (2 h, 6 h, 1, 3, 7, and 14 days). 5 mL of fresh Milli-Q water was added into the tubes again and the immersion continued.

1 mL of the immersed solution of each sample was transferred into a screw cap tube (Sarstedt, Nümbrecht, Germany) and mixed with 1 mL of total ionic strength adjustment buffer II solution (TISAB II solution, Sigma-Aldrich, Søborg, Danmark). Then, fluoride ion release was measured using a pH/ISE meter (AccumetTM AB250 Benchtop, Fisher Scientific GTF AB, Göteborg, Sweden) and a OrionTM Fluoride Electrode (Fisher Scientific GTF AB, Göteborg, Sweden). Five samples were measured for each group.

### 2.8 Cytotoxicity assay

Human TERT-immortalized gingival fibroblasts (hGFBs, CRL-4061, ATCC) were cultured in a complete cell culture medium containing Dulbecco’s modified eagle medium (DMEM, Gibco) with 10% fetal bovine serum (FBS, Gibco) and 1% penicillin/streptomycin (Gibco) in a humidified atmosphere of 5% CO_2_ at 37°C. The complete cell culture medium was changed every other day. When reaching 80%–90% confluency, hGFBs were harvested with 0.25% trypsin/EDTA (Gibco) for 2 min and passaged.

100 μL of hGFBs suspension containing 5,000 cells in complete cell culture medium were seeded in one well of a 96-well plate. After the plate was pre-incubated for 24 h in a humidified incubator, the complete cell culture medium was changed according to the experimental set-ups: the standard group, the PEO-0 group, the PEO-10 group, the PEO-20 group, and the PEO-30 group. 3 samples were tested for each group. For the standard group, the complete cell culture medium was used. For the other 4 groups, the medium was harvested after the samples (1.5 cm^2^) were immersed in 500 μL of complete cell culture medium at 37°C for 24 h, and this medium was named as the extract medium. After pre-incubation, the medium was changed into 100 μL of complete cell culture medium each well for the standard group, and 100 μL of extract medium each well for the experimental groups. The plate was incubated for 1 and 3 days in the humidified incubator (MCO-18AIC, Sanyo Electric Biomedical Co., Ltd., Osaka, Japan). At the indicated time points, 10 μL of cell counting kit-8 (CCK-8, Sigma-Aldrich) solution was added to each well of the plate. Then the plate was incubated for 2 h in the humidified incubator before being measured the absorbance at 450 nm using a microplate reader (Multiskan Go, Thermo Fisher Scientific, Vantaa, Finland). Cell viability of each experimental group was calculated according to the following equation.

$$Cell\;viability\left(\%\right)=\frac{A-B}{C-B}\times 100\%$$ (1)

In Eq. 1, A, B and C are the absorbance of the experimental groups, the blank well and the standard group.

### 2.9 Antibacterial assay

Films were cut into pieces with a total area of 1 cm^2^ in each group and disinfected by ultraviolet light for 40 min. Then the film of each group was co-cultured with *S. mutans* in 1 mL of phosphate buffer saline (PBS, Gibco) at 4°C for 24 h on an orbital shaker (DOS-10L, ELMI Ltd., Riga, Latvia). The density of *S. mutans* was 5 × 10^4^ colony forming units (CFU)/mL. *S. mutans* in PBS without any film was set up as the positive control group. After co-culture, the extracts were diluted at 1:10 with PBS, and 100 μL of the diluted extract was spread on the blood agar plates (Columbia blood agar base from Neogen, NCM 2023, Lansing, United States) with 5% defibrinated horse blood in triplicate. The blood agar plates were incubated in a CO_2_ incubator (MCO-17AIC, Sanyo Electric Biomedical Co., Ltd., Osaka, Japan) at 37°C for 24 h. After incubation, the number of CFU was assessed and the antibacterial ratio was calculated according to the following equation (Guo et al., 2018).

$$Antibacterial\;ratio\;\left(\%\right)=\frac{B-A}{B}\times 100\%$$ (2)

In Eqs. 2, A and B indicate the average value of CFU on the plates of the experiment groups and the positive control group.

### 2.10 Statistical analysis

Before performing statistical analysis, data were run for normality distribution. All data were presented as mean ± standard deviations and analyzed with One-way Analysis of Variance (ANOVA) followed by Tukey’s *post hoc* test for pair-wise comparison. The level of significance was set at *p* < 0.05.

## 3 Results and discussion

### 3.1 Structure and composition of the samples

The molecular weight of PEO has a significant impact on its solubility, viscosity, and mechanical properties. In this study, we noted that PEO with a molecular weight less than 2 × 10^5^ is mechanically incompatible with the hard polymer matrix, thus may influence the mechanical properties of the resultant polymer blend, whereas PEO with a molecular weight above 9 × 10^5^ results in significantly higher viscosity, thus increasing the difficulty of the mixing process (Apicella et al., 1993). Therefore, PEO with a molecular weight of 6 × 10^5^ was chosen in this study.

In the preliminary test, when 0.1 g of NaF was chosen, referring to 10 g of PMMA, the fluoride-ion-release concentration of the PEO-0, PEO-10 and PEO-20 groups could not be detected by the ISE meter until 3 days. When 0.2 g of NaF was chosen, the fluoride-ion-release concentration of the PEO-0 and PEO-10 group cannot be detected until 1 day. When 0.3 g of NaF was chosen, the fluoride-ion-release concentration at any time within a 14-day immersion period can be detected by the ISE meter. Furthermore, the accumulated fluoride ion release from the blend films after a 14-day immersion period was within the optimal range (5–80 ppm) to prevent caries formation (PEO-0 group: 5.39 ppm, PEO-10 group: 29.98 ppm, PEO-20 group: 27.64 ppm, PEO-30: 67.86 ppm) (Dijkman and Arends, 1992; Dijkman et al., 1993; Wiegand et al., 2007). It can be anticipated that when 0.4 g of NaF was chosen, the accumulated fluoride ion release of the PEO-30 group would exceed the range of optimal fluoride ion concentration (80 ppm). Therefore, 0.3 g of NaF was selected in this study, referring to 10 g of PMMA for all four groups.

**FIGURE 1:**
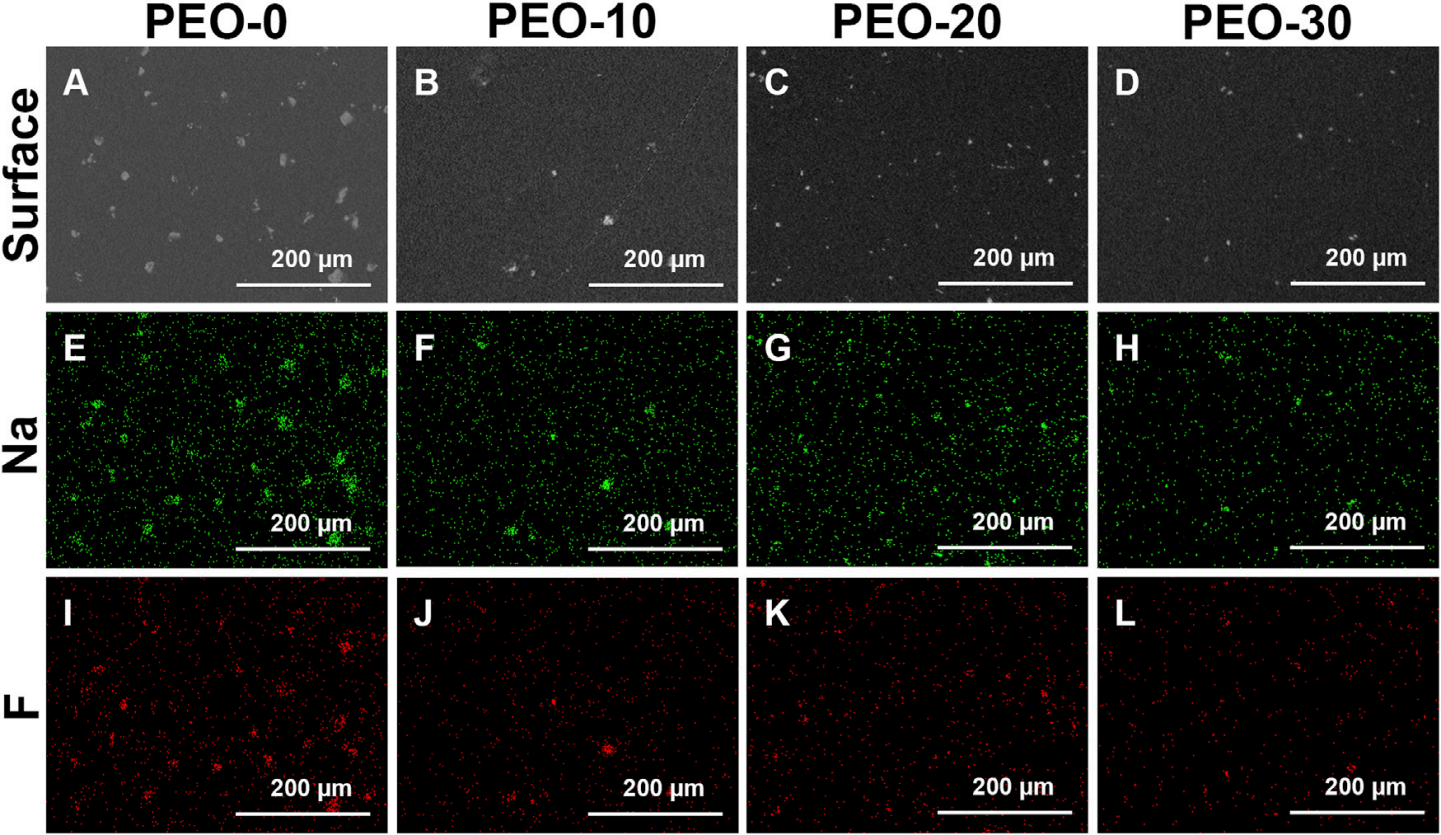
Surface morphologies of the PEO-0 group **(A)**, the PEO-10 group **(B)**, the PEO-20 group **(C)** and the PEO-30 group **(D)** characterized by SEM. Sodium distribution (green dots) of the PEO-0 group **(E)**, the PEO-10 group **(F)**, the PEO-20 group **(G)** and the PEO-30 group **(H)** obtained by EDS mapping. Fluoride distribution (red dots) of the PEO-0 group **(I)**, the PEO-10 group **(J)**, the PEO-20 group **(K)** and the PEO-30 group **(L)** obtained by EDS mapping.

Figures 1A–D show the surface morphologies of four groups, which were captured by the SEM. White spots were observed on the black background for all four groups. But we noted the distinct difference among others is that the white spots on the PEO-0 group (Figure 1A) were much more and much larger than those on the PEO-added groups (Figures 1B–D). Comparing the PEO-10 group (Figure 1B) and the PEO-20 group (Figure 1C), the white spots in the PEO-30 group (Figure 1D) were even smaller. Moreover, there were fewer white spots in PEO-30 group (Figure 1D) than in PEO-20 group (Figure 1C).

Next, we performed the EDS analysis in mapping mode to investigate the element composition of the white spots observed by SEM. Figures 1E–L show the element distribution of different groups. Specifically, Figures 1E–H display the sodium (Na) distribution in green and Figures 1I–L display the fluoride (F) distribution in red. The distribution of Na and F was highly consistent with the distribution of white spots shown in Figures 1A–D, so it is concluded that the white spots on the surface of all four groups are NaF clusters. And it could be seen more clearly in Figures 1E–L that compared with the samples without PEO (Figures 1E, I), the size of the clusters on the surface of the samples added with PEO (Figures 1F–H, J–L) was smaller and more dispersed.

**FIGURE 2:**
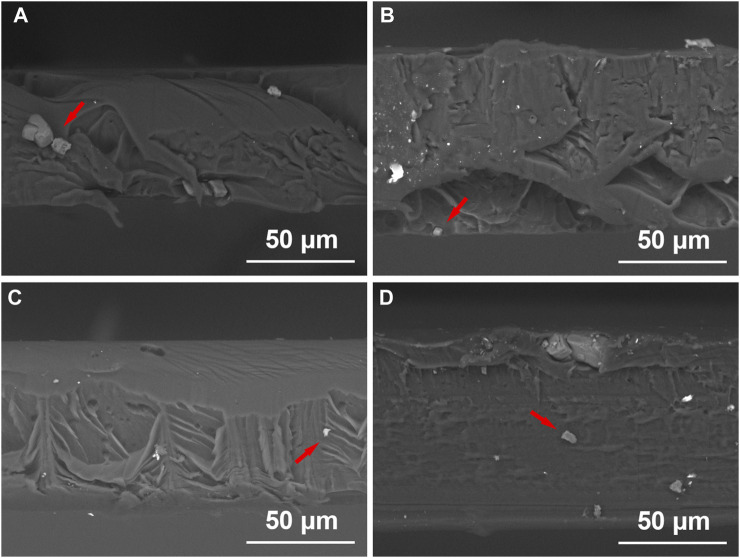
Cross-sectional images of the PEO-0 group **(A)**, the PEO-10 group **(B)**, the PEO-20 group **(C)** and the PEO-30 group **(D)** obtained by SEM. NaF clusters were marked by red arrows.

Obviously, without adding PEO, NaF formed large clusters that sit on the surface of films as shown in Figures 1A, E, I. Introducing PEO in PMMA could significantly reduce the size of NaF clusters on the film surface as shown in Figures 1B–D, F–H, J–L. It was reported that PEO is very likely to penetrate and disperse into the PMMA bulk (Apicella et al., 1993), so the next question is if NaF could also disperse well into PMMA by the presence of PEO. To investigate the longitudinal distribution of NaF inside PMMA films, the cross-sectional morphologies of the samples were conducted by the SEM, as shown in Figure 2. In Figure 2, the white spots, confirmed by EDS to be NaF clusters, were marked by red arrows. It clearly showed that the NaF clusters were not only sitting on the surface but also able to penetrate into PMMA bulk despite whether PEO was added. But we wish to call attention that the NaF clusters blended inside the PEO-0 group (Figure 2A) were much larger than those inside the PEO-added groups (Figures 2B–D), which was consistent with our observation from surface characterization by SEM in Figure 1, that NaF clusters on the surface of the PEO-0 group were much larger than other groups. Therefore, involving PEO in PMMA not only improved the dispersion of NaF clusters on the surface but also enhanced the penetration and longitudinal dispersion of NaF clusters inside PMMA films.

Since the PEO-added groups contain two polymers, it is also important to investigate whether PMMA and PEO can be blended uniformly. It was worth noting that there was no obvious phase separation in the surface morphology of the samples (Figures 1A–D), and there is no delamination in the cross-sectional images (Figure 2). AFM was also used to analyze whether there is phase separation which is shown in Supplementary Figure S1. It showed relatively high surface roughness over 100 nm based on the scanned area in Supplementary Figures S1A–D. Moreover, in the images of phase distribution, a clear phase separation could be observed as there were bright areas and dark areas in Supplementary Figures S1E–H.

However, what these two phases are cannot be determined from AFM images alone. Therefore, XRD measurements were performed on all samples as shown in Supplementary Figure S2. Pure PEO exhibits two XRD diffraction peaks at 2*θ* around 19.2°–19.3° and 23.3°–23.5°, while none of them were detected (Polu and Rhee, 2016; Yap et al., 2019). There were two wide bands (broad peaks) with remarkable intensities at 2*θ* of 7.66° and 13.67° in the spectrum of the PEO-0 group, which corresponded to PMMA since pure PMMA is amorphous (Chen et al., 2018; Yap et al., 2019). The intensities of the wide bands decreased in the PEO-10, PEO-20, and PEO-30 groups because of the addition of PEO. Furthermore, the XRD pattern shows two clear peaks, one with high intensity at 2*θ* around 38.77° and the second one with low intensity at 2*θ* around 56.16°, corresponding to (2 0 0) and (2 2 0) crystalline planes in the NaF crystal (Zhang Y. J. et al., 2021; Jiang et al., 2022). Above all, the phase separation was between PMMA/PEO and NaF, and there was no phase separation between PMMA and PEO (Shi and Han, 2012).

We further employed FTIR spectra to investigate the uniformity of the polymer blends by characterizing bonding information. As Supplementary Figure S3 shows, we identified the bands at 2,951, 1,732 and 1,448 cm^-1^ were assigned to C-H stretching, C=O stretching and C-H bending from PMMA, respectively (Park et al., 2006; Rouein et al., 2022). While, two other characteristic bands were found at 959 and 833 cm^-1^, which were attributed to CH_2_ asymmetric rocking motion from PEO (Reddy et al., 2016). But there was no obvious difference in either the band profiles or intensities among all four samples. It revealed that all samples owned the same functional groups, and the amounts of each functional group were close. There was also no formation of new chemical bonds, illustrating that there was no chemical reaction between PMMA and PEO. Shortly, all groups were prepared via a simple mechanical mixing process and two polymers were blended homogenously.

### 3.2 Surface properties

**FIGURE 3:**
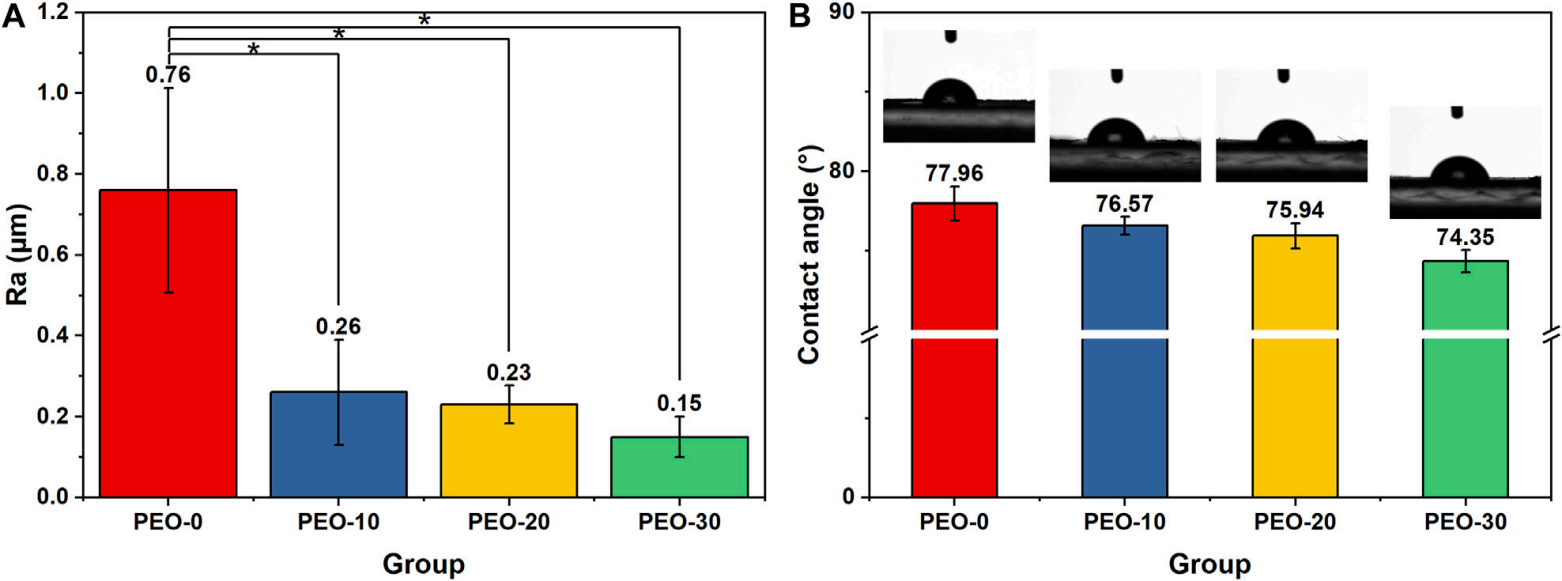
Average surface roughness obtained by surface profilometer **(A)** and the contact angle obtained by theta lite optical tensiometer **(B)** of the PEO-0 group (red), the PEO-10 group (blue), the PEO-20 group (yellow) and the PEO-30 group (green). (*, statistical difference between groups with a level of significance at *p < 0.05*).

For dental materials, the roughness and wettability of the material are important surface properties, as they relate to the adhesion of cells and bacteria to material surfaces. For this reason, we characterized the average surface roughness of the samples by a surface profilometer with a square scanning area of 5 × 5 mm^2^. Figure 3A shows that the surface roughness of the PEO-0 group is 0.76 µm. When 10% PEO was added, the surface roughness was dramatically dropped to 0.26 µm. For the PEO-20 and PEO-30 groups, the surface roughness slightly decreased to 0.23 and 0.15 µm, respectively.

We noted that the reduction trend of average surface roughness after adding PEO is in line with our observation mentioned in SEM surface scanning, that many large NaF clusters sit on the surface of the PEO-0 group (Figure 1A) while fewer and smaller NaF clusters are present on the surface of the PEO-10, PEO-20, and PEO-30 groups (Figures 1B–D). Furthermore, as confirmed by Figure 2, by introducing PEO into PMMA film, NaF can penetrate PMMA more easily and consequently, the roughness was reduced. It was reported that average surface roughness exceeding 0.4 µm can acquire retention niches for bacteria and act as shelters, consequently, a greater accumulation of plaques can be formed (Zharmagambetova et al., 2017; Condò et al., 2021). Therefore, a relatively lower surface roughness is more desirable. This reveals that another benefit of adding PEO into PMMA is that the number of bacteria adhered to the PEO-10, PEO-20 and PEO-30 groups is expected to be lower than that of the PEO-0 group. From the practical point of view, it is also important that smooth surfaces could make the splints more comfortable for the patients (Beyth et al., 2008).

The wettability of each group was measured by an optical tensiometer. Figure 3B shows the static contact angle of all samples, in which the pictures above the bars were the representative images in the tests. As shown in Figure 3B, the contact angle of the PEO-0 group was 77.96°, and the contact angle reduced after PEO was added. Among the PEO-added groups, as the amount of mixed PEO increased, the contact angle of the groups also decreased from 76.57° to 74.35°. The trend clearly indicates that hydrophilicity is increasing due to the increase of PEO content. This result confirmed our hypothesis mentioned in the Introduction, that the addition of PEO, an amphipathic polymer material, could effectively modify the surface wettability of the polymer blends. The improved wettability is expected to not only enhance the release of fluoride ions as water could spread more easily on the surface of the film but also improve the biocompatibility of the blend films. We further repeated the wettability measurement (3 times) for all groups and did not observe obvious fluctuations for all the samples, which, once again, confirmed that PEO was blended with PMMA uniformly. If the PMMA and PEO blends suffer phase-separation or form large domains, we would anticipate that the contact angle will vary greatly when the measurement is conducted at different locations.

### 3.3 Fluoride ion release

The fluoride ion release behavior was investigated in the immersion test. The fluoride-ion-release concentrations were measured by the pH/ISE meter. As we described in the Experimental sections, all samples measured in the fluoride ion release test were first immersed in Milli-Q water for 2 h to remove the NaF clusters that sit on the polymer film surface and eliminate the interference caused by the rapid surface releasing. By doing this, we anticipated the release of fluoride ions could correctly reflect the release of clusters dispersing inside the polymer films, as manifested by the cross-sectional SEM characterization in Figure 2.

**FIGURE 4:**
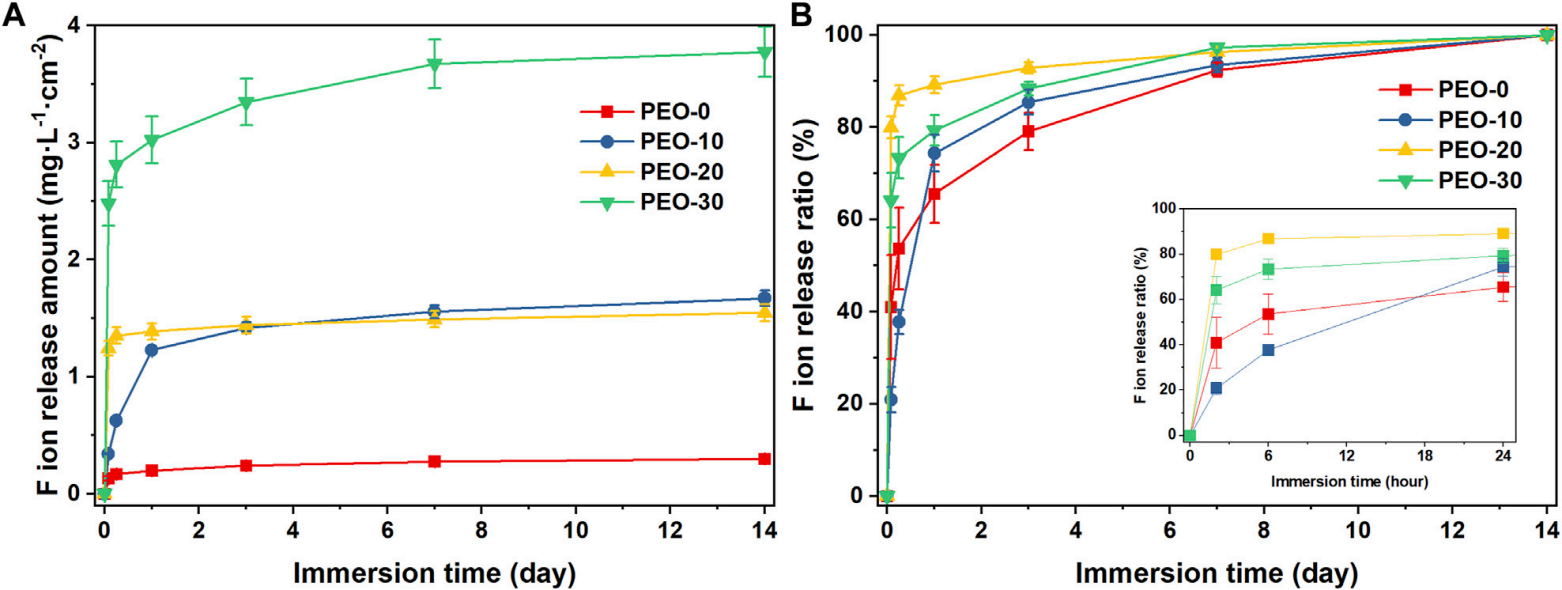
Fluoride ion release amount obtained by pH/ISE meter **(A)** and the fluoride ion release ratio calculated from the fluoride ion release amount **(B)** of the PEO-0 group (red line and square points), the PEO-10 group (blue line and circle points), the PEO-20 group (yellow line and upward triangle points) and the PEO- 30 group (green line and downward triangle points). (The figure in the bottom right of **(B)** is a partial enlargement of immersion time from 0 to 1 day).

Figure 4A displays the amount of fluoride ion release of different group samples. Over the entire testing period (14 days), the average total fluoride ion release amount of the PEO-0 group was only 0.30 mg L^-1^ cm^-2^. As mentioned in the Introduction, it was below the concentration that may cause bone fluorosis and gastric irritation, which was 4 mg/L and 0.1 mg/kg, respectively (National Research Council, 1993; Akiniwa, 1997; Arlappa et al., 2013; Yadav et al., 2019). After adding PEO, the total fluoride ion release of the samples increased significantly. Specifically, the total amount of fluoride ion release of the PEO-10 and PEO-20 groups was close, reaching 1.67 and 1.55 mg L^-1^ cm^-2^, respectively, which was ca. 5 times more than that of the PEO-0 groups. The fluoride ion release of the PEO-30 group even reached 3.78 mg L^-1^ cm^-2^, which was 12 times as large as that of the PEO-0 group. This data implied that the addition of PEO indeed provides an ion release pathway for fluoride ions from NaF clusters which disperses inside of the PMMA/PEO blend films, as we proposed earlier in the Introduction. We speculated that during the immersion process, as the water penetrates in, PEO molecules consequently absorb water and swell, causing the NaF clusters to dissolve and fluoride ions to release via the PEO molecular chain.

It is, in addition, worth noting that there was a rapid release in the initial 6 h for the PEO-20 and PEO-30 groups. The PEO-20 group released more than 1 mg L^-1^ cm^-2^ and the fluoride ion release amount of the PEO-30 group even reached more than 3 mg L^-1^ cm^-2^. After the initial rapid release, the release rate of the PEO-20 and PEO-30 groups gradually became stable. In contrast, the release of the PEO-10 group was more sustained till day 3 (0.62 mg L^-1^ cm^-2^).

This trend can be seen more intuitively in Figure 4B, which presents the fluoride ion release ratio that was calculated by dividing the cumulative amount of fluoride released at each time point by the total amount released throughout the testing period. The PEO-10 group only released 20.9% of fluoride ions in the first 6 h, and it maintained a relatively stable release rate during the first day. In comparison, the PEO-20 and PEO-30 groups released 79.9% and 64.1% of fluoride ions already in the first 6 h. After 24 h, the PEO-10 group released 74.3% of fluoride ions and then its release rate slowed down and gradually approached the release ratio of the PEO-20 and PEO-30 groups. These comparisons revealed that the PEO-10 group presents the most desirable properties as a NaF carrier and slow-release system among all four groups.

### 3.4 Cytotoxicity

**FIGURE 5:**
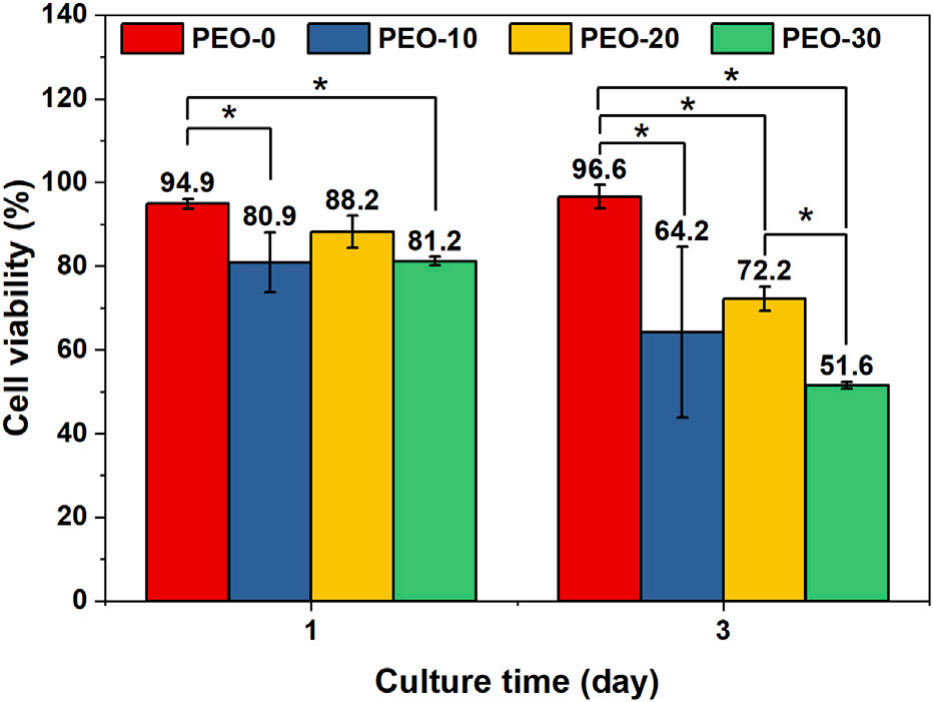
Cell viability of hGFBs cultured with the extract medium of the PEO-0 group (red), the PEO-10 group (blue), the PEO-20 group (yellow), and the PEO-30 group (green) for 1 day and 3 days. (*, statistical difference between groups with a level of significance at *p < 0.05*).

In Figure 5, CCK-8 assay showed that the cell viability of hGFBs cultured with the extract medium from the PEO-20 samples (88.2%) was slightly lower than that of the PEO-0 samples (94.9%) at day 1. Meanwhile, the cell viability of the PEO-10 samples (80.9%) and the PEO-30 (81.2%) samples was significantly lower than that of the PEO-0 samples. High cell viability (>80%) reflects low cytotoxicity (Iwasawa et al., 2013). On the other hand, because the cell viability of all samples is higher than the biocompatibility standard (70%) issued by international organization for standardization (ISO) (Bruinink and Luginbuehl, 2012; Shafiee et al., 2021), the films could be regarded as biocompatible dental materials *in vitro* at day 1.

On day 3, the cell viability of the PEO-0 samples was 96.6%, significantly higher than that of the other samples. The cell viability of the PEO-10 samples was 64.2%, indicating potential cytotoxicity, with no statistical difference from that of PEO-20 (72.2%). However, the cell viability of PEO-30 samples was reduced to 51.6%, which showed that the PEO-30 samples are cytotoxic. According to Figure 5A, the fluoride ion release amount of the PEO-30 group was two times more than that of the PEO-10 and PEO-20 groups, and the fluoride ion release amount of the PEO-10 and the PEO-20 groups was comparable. These indicate that the cytotoxicity from increased concentration of fluoride ions in the extract medium of the PEO-30 samples could be one of the possible explanations for the decreased cell viability. Above all, the biocompatibility of the PEO-0 and the PEO-20 groups is acceptable.

### 3.5 Antibacterial activity

**FIGURE 6:**
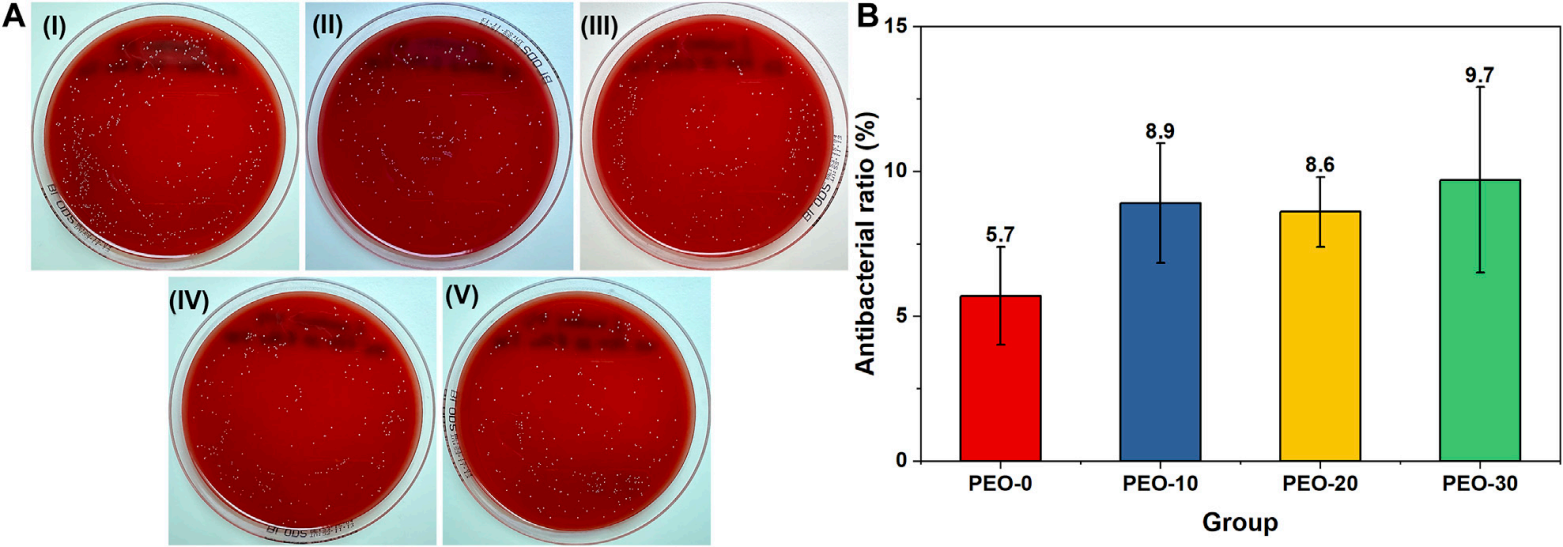
Representative images of *Streptococcus mutans* colonies (yellow dots) on blood agar plates after spreading **(A)** PBS with *Streptococcus mutans* (I), PBS with *Streptococcus mutans* and the PEO-0 group (II), PBS with *Streptococcus mutans* and the PEO-10 group (III), PBS with *Streptococcus mutans* and the PEO-20 group (IV) and PBS with *Streptococcus mutans* and the PEO-30 group (V) for 24 h at 37°C. Quantitative results of the antibacterial ratios of the PEO-0 group (red), the PEO-10 group (blue), the PEO-20 group (yellow) and the PEO-30 group (green) **(B)**.

Figure 6A showed representative images of *S. mutans* with several colonies (yellow dots) on blood agar plates. The number of colonies of each sample was compared to that of the positive control group. The quantitative result in Figure 6B showed that the antibacterial ratios of all the groups were lower than 10%. There was no significant difference between samples. Therefore, the antibacterial activity of the films can be negligible.

## 4 Conclusion

In this research, we fabricated a PMMA-based blend film with NaF and different proportions of PEO by solution casting. The surface properties, the fluoride ion release and the biological properties were investigated. With an increased ratio of PEO, NaF clusters distributed more uniformly both on the surface of and inside of polymer blend films; and the average surface roughness and water contact angle decreased. The addition of PEO effectively regulated the fluoride ion release behavior of the blend films. Among all samples, the PEO-10 samples, which consisted of PMMA, PEO and NaF with a mass ratio of 10: 1: 0.3, showed a sustained release profile of fluoride ions over 14 days. Furthermore, the amount of fluoride ion release showed a negative relationship with biocompatibility. The *in vitro* biocompatibility of the PEO-10 samples and the PEO-20 samples was acceptable. Taken together, the PEO-10 samples realized a slow release of fluoride ions with low cytotoxicity. Therefore, PMMA/PEO polymer blends together with NaF can be a safe and promising strategy to endow dental appliances with anti-cariogenicity.

## Data Availability

The original contributions presented in the study are included in the article/Supplementary Material, further inquiries can be directed to the corresponding authors.
