# Supplementary material for: Fluoride releasing in polymer blends of poly(ethylene oxide) and poly(methyl methacrylate)
Source: Front Chem. 2024 Feb 9;12:1356029. doi: 10.3389/fchem.2024.1356029 (PMC10884178; doi:10.3389/fchem.2024.1356029)
Supplement: Supplementary file 1 [file DataSheet1.docx]

Supplementary Material

Realization of Fluoride Releasing in Polymer Blends of Poly (Ethylene Oxide) and Poly (Methyl Methacrylate)

**Tianxiao Wang^1†^, Menghong Li^1†^, Ziyan Gu^1^, Chengjuan Qu^1^, Jonas Segervald^2^, Roushdey Salh^2^, Thomas Wågberg^2^, Jia Wang^2*^, Wen Kou^1*^**

**Table S1** The composition of the experimental groups.

| Group | PMMA (g) | PEO (g) | NaF (g) |
| --- | --- | --- | --- |
| PEO-0 | 10 | 0 | 0.3 |
| PEO-10 | 10 | 1 | 0.3 |
| PEO-20 | 10 | 2 | 0.3 |
| PEO-30 | 10 | 3 | 0.3 |


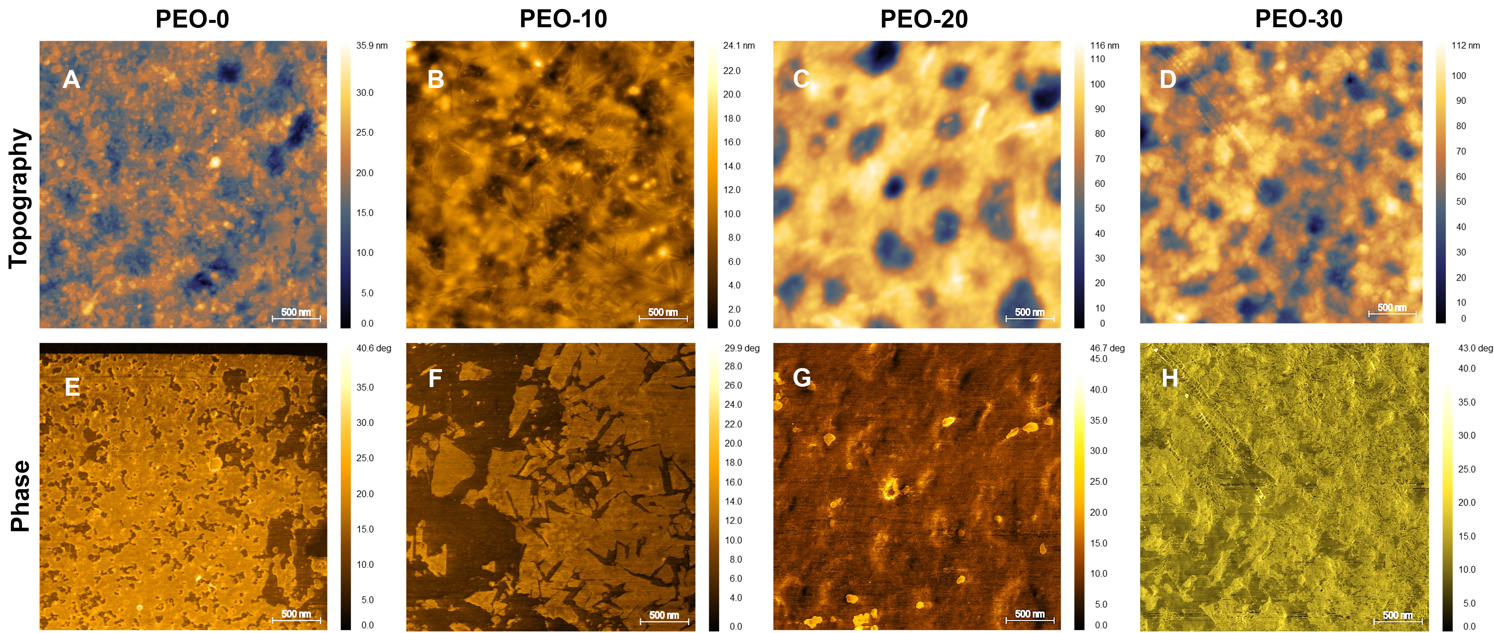


**Figure S1**
Topography images of the PEO-0 group **(A)**, the PEO-10 group **(B)**, the PEO-20 group **(C)** and the PEO-30 group **(D),** the phase distribution of the PEO-0 group **(E)**, the PEO-10 group **(F)**, the PEO-20 group **(G)** and the PEO-30 group **(H)** were characterized by AFM under taping mode. The scanning area is 3×3 µm ^2^.





**Figure S2**
XRD spectra obtained by the X-ray diffractometer of the PEO-0 group (red line), the PEO-10 group (blue line), the PEO-20 group (yellow line) and the PEO-30 group (green line). A partial enlargement of the 50- to 60-degree range is in the upper right of the figure.





**Figure S3**
FTIR spectra obtained by the FTIR spectrometer of the PEO-0 group (red line), the PEO-10 group (blue line), the PEO-20 group (yellow line) and the PEO-30 group (green line).
